# Evaluation of Choroidal Melanoma Vascularization by Color Doppler Flow Imaging: An Option for Follow-Up Tumor Control Assessment after CyberKnife^®^?

**DOI:** 10.3390/medicina57060553

**Published:** 2021-05-31

**Authors:** Cinja Kaak, Vinodh Kakkassery, Björn O. Scheef, Marco Zschoche, Felix Rommel, Guido Hildebrandt, Steffen Emmert, Christian Junghanß, Rudolf F. Guthoff, Anselm M. Jünemann, Uwe Walter

**Affiliations:** 1 Department of Ophthalmology, University Medical Center Rostock, 18057 Rostock, Germany; cinjaeileen@hotmail.de (C.K.); b_scheef@gmx.de (B.O.S.); marco.zschoche@gmail.com (M.Z.); rudolf.guthoff@med.uni-rostock.de (R.F.G.); anselm.juenemann@outlook.de (A.M.J.); 2 Department of Ophthalmology, University of Lübeck, 23538 Lübeck, Germany; felix.rommel@uksh.de; 3 Department of Radiotherapy, University Medical Center Rostock, 18059 Rostock, Germany; guido.hildebrandt@med.uni-rostock.de; 4 Clinic and Polyclinic for Dermatology and Venereology, University Medical Center Rostock, 18057 Rostock, Germany; steffen.emmert@med.uni-rostock.de; 5 Department of Oncology, University Medical Center Rostock, 18057 Rostock, Germany; christian.junghanss@med.uni-rostock.de; 6 Department of General and Pediatric Ophthalmology Service, Medical University of Lublin, 20-079 Lublin, Poland; 7 Department of Neurology, University Medical Center Rostock, 18147 Rostock, Germany

**Keywords:** choroidal melanoma, CyberKnife, plaque brachytherapy, local tumor control, color Doppler flow imaging, tumor vascularization

## Abstract

*Background and Objectives*: Thus far, tumor control for choroidal melanoma after teletherapeutic radiation is clinically difficult. In contrast to brachytherapy, the tumor height does not necessarily have to shrink as a result of teletherapy. Therefore, the objective of this study was to evaluate tumor vascularization determined by color Doppler flow imaging (CDFI) as a possible approach for monitoring the therapy response after teletherapy of choroidal melanoma. *Materials and Methods*: A single-center retrospective pilot study of 24 patients was conducted, all of whom had been diagnosed with choroidal neoplasm, treated and followed up. Besides tumor vascularization, the following parameters were collected: age, gender, tumor entity, location, radiation dose, knowledge of relapse, tumor height, radiation-related complications, occurrence of metastases, visual acuity in logMAR. *Results*: The level of choroidal melanoma vascularization markedly decreased in all included subjects after treatment with the CyberKnife^®^ technology. Initially, the level of vascularization was 2.1 (SD: 0.76 for *n* = 10); post-therapeutically, it averaged 0.14 (SD: 0.4). Regarding the tumor apex, CDFI sonography also demonstrated a significant tumor regression (mean value pre-therapeutically: 8.35 mm—SD: 3.92 for *n* = 10; mean value post-therapeutically: 4.86 mm—SD: 3.21). The level of choroidal melanoma vascularization declined in the patient collective treated with ruthenium-106 brachytherapy. The pre-therapeutic level of vascularization of 2 (SD: 0 for *n* = 2) decreased significantly to a level of 0 (mean: 0—SD: 0). The tumor height determined by CDFI did not allow any valid statement regarding local tumor control. In contrast to these findings, the patient population of the control group without any radiation therapy did not show any alterations in vascularization. *Conclusions*: Our data suggest that the determination of the tumor vascularization level using CDFI might be a useful and supplementary course parameter in the follow-up care of choroidal melanoma to monitor the success of treatment. This especially applies to robot-assisted radiotherapy using CyberKnife^®^. Further studies are necessary to validate the first results of this assessment.

## 1. Introduction

Choroidal melanoma is the most common primary malignant tumor of the eye in adults [1,2]. In contrast to cutaneous melanoma, the incidence rate of choroidal melanoma is stable, ranging from 1.3 to 8.6 cases per 1,000,000 in the US and Europe [3,4]. With a 5-year mortality risk of up to 40%, it is the most common cause of death in the field of ophthalmology [5]. Local treatment of choroidal melanoma consists of either globe-preserving therapies or enucleation [6]. Eye-preserving treatment options include brachytherapy using a ruthenium-106 or iodine-125 applicator and teletherapeutic therapy regimes such as proton beam radiation, Gamma Knife radiosurgery and robot-assisted radiotherapy (CyberKnife^®^) [7,8]. In the subsequent evaluation of the therapeutic effect, the post-therapeutic investigation of choroidal melanomas focuses primarily on the course of the tumor height ascertained with the aid of sonography and fundoscopic findings. Since the absence of scarring and an initial decrease in prominence is not unusual after a teletherapeutic procedure, the criteria for therapeutic response that are common in the course of aftercare cannot be applied without restrictions and should possibly be supplemented. The objective of this study is the comparison of the pre- and post-therapeutic tumor vascularization of choroidal melanomas determined by color Doppler flow imaging (CDFI) sonography after radiation therapy. This study focuses on robot-assisted radiotherapy (CyberKnife^®^) but also reports on a small subgroup that received ruthenium-106 brachytherapy. Patients who received conservative therapy without further radiation therapy served as a control group. Furthermore, the objective of this study was to evaluate tumor vascularization determined by CDFI as a possible approach for monitoring the therapy response after teletherapy of choroidal melanoma and to compare treatment options regarding local tumor control, visual acuity and the complications resulting from radiotherapy.

## 2. Materials and Methods

### 2.1. Study Design

A retrospective study was conducted with 24 patients, all of whom had been diagnosed with choroidal neoplasm, treated and followed up between July 2005 and December 2017 in the Department of Ophthalmology and the Department of Radiotherapy at the University Medical Center Rostock. For the elicitation of the patient data, the patient files were evaluated, including the medical reports, operation reports, ultrasound findings and radiation concepts. Furthermore, the values recorded in the course of the last ophthalmological and imaging control diagnostics could be independently collected and analyzed.

This research project was approved by the ethics committee of the Medical Faculty of the University of Rostock (14 June—A 2017-0077). Furthermore, the implementation of this study is in accordance with the Declaration of Helsinki.

### 2.2. Patients

For the present study, the data of all patients were recorded in whom a choroidal tumor was diagnosed in the above-mentioned period and a suitable therapy regimen was administered depending on the tumor entity. Patients needed to be followed up for at least two times to be included in this study. According to the chosen treatment method, patients were stratified into one of these therapy groups: Eleven of the twenty-four patients received robot-assisted radiotherapy using CyberKnife^®^. Four of the patients were treated with brachytherapy according to the prominence of the mass. Five individuals served as a control group without any radiation therapy. An additional total of four patients had to be enucleated during this study to treat the extensive cancerogenic disease in the eye. The neoplasms of the patient collective that received radiation therapy using CyberKnife^®^ or ruthenium-106 brachytherapy were all identified as choroidal melanomas. This also applies for those patients who had to be enucleated during the follow-up period. In the control group, one tumor was characterized as a choroidal melanoma and three as choroidal hemangiomas. The entity of one neoplasm remained uncertain, meaning that it was classified as a choroidal prominence of unknown origin.

### 2.3. Clinical Data

The following demographic and clinical parameters were collected: age, gender, tumor entity, tumor location, radiation dose, radiation-related complications, occurrence of a relapse, tumor height, occurrence of metastases and the visual acuity in logMAR. The ultrasound tumor measurements were performed using a standardized A- and B-scan echography.

### 2.4. Color Doppler Flow Imaging

Using CDFI, the size of the choroidal tumor as well as the level of vascularization was assessed [9,10]. All investigations were performed by an experienced sonographer (UW) blinded to the ophthalmological and radiological data of the patients who were assessed by the ophthalmologist on the same day only after the ultrasound investigation. Ocular CDFI sonography was performed using a high-end ultrasound system (Acuson Antares, Siemens, Erlangen. Germany) equipped with a 7.5-Mhz transducer. According to the regulations of the Center for Devices and Radiological Health, the B-mode ultrasound energy output was set at a mechanical index of <0.3 and a thermal index for the crabiao bone of <1.0 [9]. Ocular CDFI was applied only for short periods of <2 min, and with the energy output set at a mechanical index of <0.7. Ultrasound application through the eye lens was avoided whenever possible. The levels of tumor vascularization were classified visually on a semi-quantitative scale as published before (Table 1) [9]. In addition, CDFI flow curves of intra-tumor arteries were acquired if at least a low vascularization was present, and the following flow parameters were calculated by the ultrasound system software: resistance index, pulsatility index.

**Figure 1:**
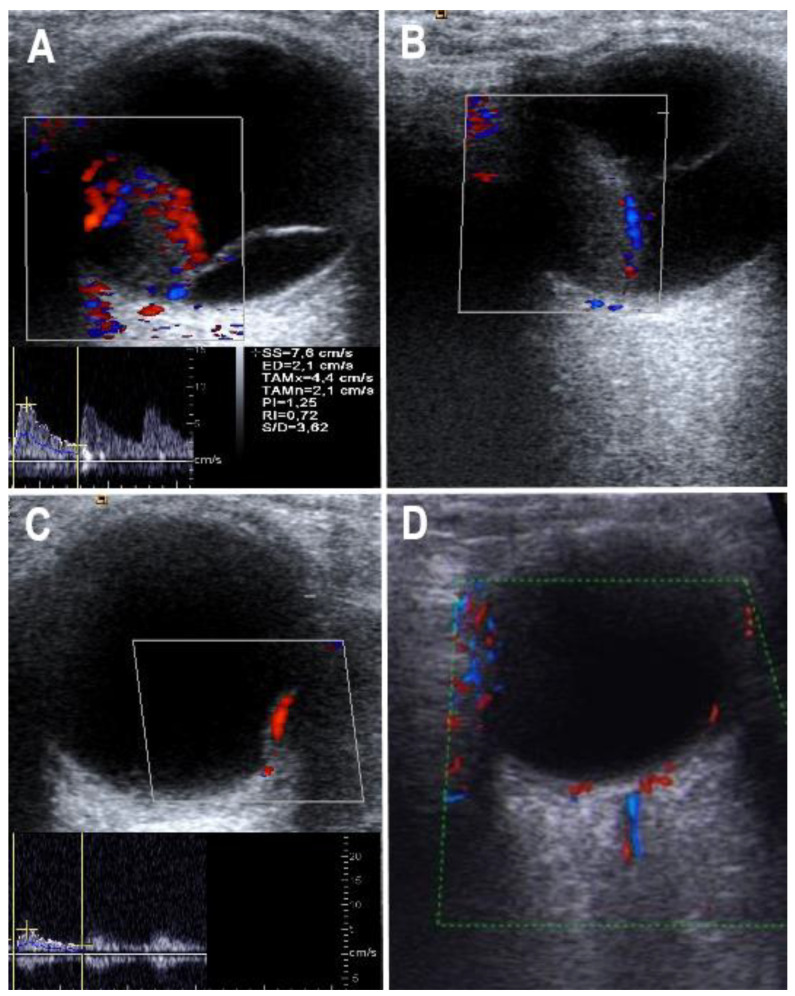
Color Doppler flow imaging of choroidal melanoma before and after therapy. (**A**) Patient with a large choroidal melanoma at inferior nasal location of left eye. Note the high degree of intra-tumor vascularization (level 3). The insert in the lower part shows the CDFI flow curve and indices of a prominent intra-tumor artery. (**B**) Choroidal melanoma 3 months after CyberKnife^®^ therapy in the same patient as shown in (**A**). Note the complete loss of intra-tumor vascularization (level 0), with visibility only of sub-retinal vessels. (**C**) Patient with a small choroidal melanoma at temporal location of right eye. Note the prominent singular intra-tumor artery (vascularization level 2). The insert in the lower part shows the CDFI flow curve of the intra-tumor artery. (**D**) Choroidal melanoma 12 months after CyberKnife^®^ therapy in the same patient as shown in (**C**). Note the nearly complete loss of intra-tumor vascularization (level 1), with visibility only of a low-flow intra-tumor artery.

All sonographic assessments were carried out prior to the tumor radiation therapy, 1 and 2 years after radiation and thereafter at the final follow-up, which does not represent an exactly uniform period of time [11,12,13,14]. Representative ultrasound images are shown in Figure 1.

### 2.5. Statistical Analysis

Statistical calculations were performed using SPSS (IBM, Version 25.0, Chicago, IL, USA). Quantitative variables were summarized as mean and standard deviation and qualitative variables as frequency and percentage. Normal distribution was checked by the Shapiro–Wilk test.

Due to a standard or t-distribution in our study groups, the paired *t*-test was used to compare between baseline and follow-up parameters of the same eye. To test the statistical significance of the difference between two mean values recorded for the control points, the *t*-test for connected samples was used. The level of significance was set at *p* < 0.05.

## 3. Results

### 3.1. Patients

Demographics are reported in Table 2.

### 3.2. Course of Tumor Vascularization during Treatment

**Figure 2:**
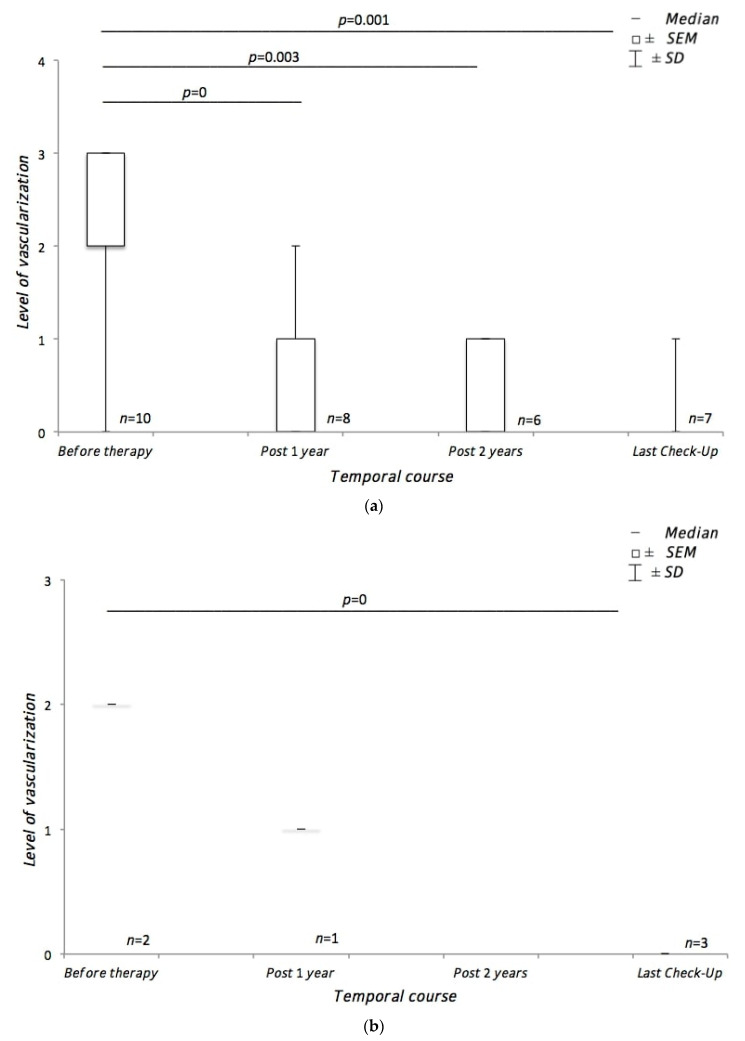
Course of tumor vascularization during treatment. (**a**) shows the course of choroidal melanoma vascularization using CDFIy in patients treated with CyberKnife^®^. (**b**) shows the course of choroidal melanoma vascularization using CDFI in patients treated with ruthenium-106 brachytherapy.

In the subgroup of patients treated by radiotherapy using CyberKnife^®^, the mean level of choroidal melanoma vascularization was 2.1 ± 0.76. At final follow-up, the tumor vascularization decreased to a mean level of 0.14 ± 0.4 (*p* = 0.001) (Figure 2).

In all patients with vascularization level 3 or 2 prior to CyberKnife^®^ therapy, at the first follow-up investigation after 3 months, vascularization was decreased, and after 6 months, usually the individual minimum of vascularization was reached, with stability during the further follow-up. The CDFI sonography indices decreased continuously after therapy (decrease in mean pulsatility index prior to therapy until the 12th month after therapy: from 1.3 to 0.9; in mean resistance index: from 0.7 to 0.4). However, there was some intra-individual variation from visit to visit, and the individual CDFI indices could no longer be assessed if vascularization level 0 was achieved. Therefore, the vascularization level assessed on CDFI was regarded as the most suitable ultrasound parameter for long-term follow-up.

The subgroup treated with brachytherapy had a mean level of choroidal melanoma vascularization of 2 ± 0. In the course of the last check, the mean level of vascularization was 0 ± 0 (*p* = 0) (Figure 2).

The patient population of the control group, which received conservative therapy without any treatment, showed a mean vascularization level of 1.8 ± 1.1. Post-therapeutically, the mean level of vascularization was 1.6 ± 1.14. No significant change in tumor vascularization could be documented (Supplemental Figure S1).

### 3.3. Course of Tumor Height during Treatment

**Figure 3:**
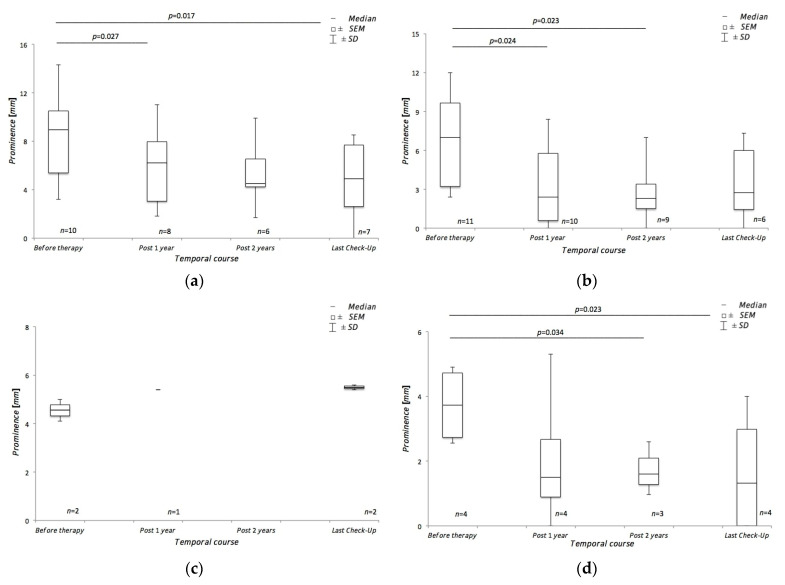
Course of tumor height during treatment. (**a**) shows the course of tumor prominence during treatment using CDFI in patients treated with CyberKnife^®^. (**b**) The figure shows the course of tumor prominence during treatment using bulbar sonography in patients treated with CyberKnife^®^. (**c**) The figure shows the course of tumor prominence during treatment using CDFI in patients treated with ruthenium-106 brachytherapy. (**d**) The figure shows the course of tumor prominence during treatment using bulbar sonography in patients treated with ruthenium-106 brachytherapy.

In the patient group who subsequently received robot-assisted radiotherapy using CyberKnife^®^, the mean tumor height determined by CDFI was 8.35 ± 3.92 pre-therapeutically. Following the treatment, the last control examination showed a mean visible tumor prominence of 4.86 ± 3.21 mm (*p* = 0.017). As part of the initial diagnostics, bulbar sonography revealed a mean tumor prominence size of 6.55 ± 3.66 mm in this patient collective. Post-therapeutically, the mean value was 3.48 ± 3.0. (Figure 3).

The group of patients who subsequently received brachytherapy using a ruthenium-106 applicator showed a mean tumor height of 4.55 ± 0.64 mm determined by CDFI. Post-therapeutically, the mean value of the tumor prominence could be documented as 5.5 ± 0.14 mm. This result could not achieve statistical significance. The transbulbar sonography, which was also performed pre-therapeutically, collected a mean value of 3.735 ± 1.22 mm in the corresponding patient group. After the end of the treatment, the final control showed a mean tumor prominence of 1.66 ± 2.0 (*p* = 0.023) (Figure 3).

Pre-therapeutically, the tumor height determined by CDFI in those patients who received a conservative treatment regimen free of radiation therapy had a mean value of 3.42 ± 1.43 mm. At final follow-up, the mean tumor prominence was 3.56 ± 1.41mm. Bulbar sonography showed a mean tumor prominence of 2.53 ± 0.47 mm before therapy. The final examination revealed a mean value of 1.81 ± 1.11 mm. Neither the results of CDFI nor those of bulbar sonography could achieve any statistical significance (Supplemental Figure S2).

## 4. Discussion

### 4.1. Course of Choroidal Melanoma Vascularization during Treatment

In terms of the pre- and post-therapeutic characteristics of tumor vascularization, we were able to detect a decrease in choroidal melanoma vascularization for all eyes which received the robot-assisted radiotherapy using CyberKnife^®^. Additionally, we found consistently low levels of vascularization in only one choroidal melanoma, while tumor vessels were completely absent in the other cases in the final examination, which was performed between three and six years after the end of treatment. The described result turned out to be statistically significant and was taken as a sign of therapeutic success. Thus far, data on CyberKnife^®^ treatment is limited to an extremely small extent and cannot be equated with the existing collection of studies of other established therapy methods.

Agreeing with our results, a single case report relating to another tumor entity, a papillary serous carcinoma of the peritoneum, by Jen-Min Su et al. showed a decrease in tumor blood flow following robot-assisted radiotherapy using CyberKnife^®^. However, the follow-up period in this case was only six months [15].

Supportive of this, our subgroup of patients treated by ruthenium-106 brachytherapy also showed a significant decrease in choroidal melanoma vascularization. A non-representable tumor blood flow was recorded in 100% of the persons considered. The rapidity of diminution of vascularization is also comparable to that of the subgroup described above. Proniewska-Stretek et al. reported on a similar decrease in choroidal melanoma vascularization of all included patients (*n* = 15) in their analysis [16]. A study published by Wolff-Kormann et al. showed a decrease in the number of tumor vessels in choroidal melanoma in 95% of the study population (*n* = 20) to the same extent. In contrast to our study, patients’ follow-up was only six months [17].

Unlike in our study groups treated with radiotherapy, the patient population of our control group, which received no radiation therapy, did not show any significant alterations in the level of vascularization.

Overall, the subjects who received robot-assisted radiotherapy using CyberKnife^®^ showed a post-therapeutic reduction in vascularization as an expression of therapeutic success. Even if a direct comparison is not possible, the brachytherapy subgroup also showed a decrease in vascularization and thus supports the findings of the larger group that received CyberKnife^®^. Compatible with this, the control group showed no significant change in vascularization.

In conclusion, the results of both our analyzed subgroups confirm the usefulness of CDFI for monitoring the therapeutic effect and thus rank among the results of other studies. Further, the pulsatility index and resistance index were decreased after radiation therapy. However, follow-up studies with larger numbers of patients should be performed to confirm our findings. In this context, it should also be clarified whether tumor vascularization can be used to identify, in advance, the extent to which a successful treatment is possible or not.

### 4.2. Course of Choroidal Melanoma Height during Treatment

Our patient collective, which was treated using CyberKnife^®^, showed a sonographic decrease in tumor height during the follow-up period in all study participants. Compatible with the results we collected, Muacevic et al. reported a local tumor control rate of 100% in their pilot study with a small sample size of seven test individuals that had been diagnosed with choroidal melanoma. Furthermore, their follow-up period was only 6 to 22 months [18]. The study subsequently performed by Eibl-Lindner et al. included a larger study population of 217 patients with choroidal melanoma. The follow-up extended over a period of five years. The results obtained in this context indicate decreased tumor control compared to our data. After three and five years, respectively, 87% and 71% of all test persons showed local tumor control [19].

Apart from bulbar sonography, we regularly determined the maximum tumor height by using CDFI as part of the follow-up. Regarding the primary and last checkpoint, which corresponds to a period of three to six years, it showed a statistically significant tumor regression in all of the subjects and thus supports the findings obtained with the help of conventional ultrasound. To the best of our knowledge, this is the first study reporting CDFI sonography as a suitable method for monitoring local tumor control of choroidal melanomas.

The entirety of our study group that was treated using ruthenium-106 brachytherapy demonstrated a significant regression in the tumor height using bulbar sonography. Damato et al. were able to show a matching tumor control rate of 99% after two and 97% after seven years of follow-up. Their patient collective consisted of a total of 458 patients diagnosed with choroidal melanoma [20]. The strict selection criteria that were applied in the trial by Damato et al. unexpectedly match our patient data. This should be considered as one of the reasons for the agreement of the results. In contrast to our findings, studies that include choroidal melanomas of the T3 stage show significantly lower tumor control rates. Performing assessment under these conditions, Rouberol et al. reported on local tumor control in 78.3% of a total of 213 patients after five years of follow-up [21].

We additionally determined local tumor control using CDFI in our study. For the patient collective treated with brachytherapy, however, this imaging method could neither demonstrate a significant decrease in the tumor prominence nor make a valid statement about local tumor control. We saw the reason for this in the insufficient data collected in around 50% of the patients and not the examination method used. Consistent with this, Proniewska-Shretek et al. analyzed CDFI as a qualified method for monitoring the progression of choroidal melanomas after brachytherapy. The study consisted of a sample size of 15 patients [16].

By way of contrast, neither the results of CDFI nor those of bulbar sonography demonstrated any significant change regarding the tumor prominence in our control group.

In conclusion, apart from the primary results, that confirm the usefulness of CDFI for monitoring the therapeutic effect, our results additionally suggest CDFI as an alternative to conventional bulbar sonography in determining the tumor height. To the best of our knowledge, there have not been any previous studies on this, especially regarding robot-assisted radiotherapy using CyberKnife^®^.

## 5. Limitations

Due to the retrospective study design, potential disadvantages must be considered. On the one hand, the patient groups have neither a congruent size of the number of patients nor an identical time of follow-up. The circumstance that the follow-up protocol could not always be fully adhered to is another limiting factor. On the other hand, due to the selected study character, biases and incompleteness regarding the collection of data cannot be ruled out. Apart from the study type, the overall volume of the patient collective should also be viewed as limiting. Furthermore, for better comparability, CDFI was performed using the same high-end ultrasound system (Acuson Antares, Siemens, Erlangen, Germany) during the entire study period, although better equipment was already available on the market.

## 6. Conclusions

The present study results suggest the potential of CDFI in the pre- and post-therapeutic diagnosis of choroidal melanomas. Therefore, the determination of the level of vascularization may offer a new dimension for assessing the success of radiation therapy treatments. This applies, in particular, to teletherapy, especially robot-assisted radiotherapy using CyberKnife^®^, as a result of which the absence of scarring and a decrease in tumor prominence is not unusual and a statement on the therapeutic effect cannot always be made with certainty. As part of our study, CDFI may also be an alternative to conventional sonography in determining tumor prominence. Despite the promising results, thus far, they can only be used as a guide and should be further evaluated.

## Figures and Tables

**Table 1:** Classification of the tumor vascularization levels on CDFI [9].

0	No vascularization
1	Low vascularization (a small internal artery can be visualized)
2	Moderate vascularization (two to three internal arteries can be visualized)
3	Strong vascularization (paying, partly strong internal arteries can be shown)

**Table 2:** Demographics and clinical data of the enrolled patients treated by either ruthenium-106 brachytherapy or robot-assisted radiotherapy using CyberKnife^®^.

	Ruthenium-106 Brachytherapy	Robot-Assisted Radiotherapy Using CyberKnife^®^
Number of patients	4	11
Age at diagnosis	Ø 67.5 ± 12.13 years	Ø 62 ± 13.68 years
Gender ♀/♂	4:0	2:9
Tumor entity	100% Choroidal melanoma	100% Choroidal melanoma
Tumor localization right/left	3:1	4:7
Tumor apex prominence at diagnosis	Sonography: Ø 3.735 ± 1.22 mm CDFI: Ø 4.55 ± 0.64 mm	Sonography: Ø6.55 ± 3.66 mm CDFI: Ø 8.35 ± 3.92 mm
Scleral radiation dose	Ø 798.5 Gy	Ø 55 Gy
Follow-up period	Ø 50.25 ± 26.19 months	Ø 43.3 ± 24.82 months
Adjuvant therapy	Transpupillary thermotherapy: in 100% Ø 2 therapy sessions	None
Metastases	None	27.3%
Recurrence	None	None

## Supplementary Materials

The following are available online at https://www.mdpi.com/article/10.3390/medicina57060553/s1, Supplementary Figure S1: Course of tumor vascularization using CDFI in the patient collective of the control group, Supplementary Figure S2a: Course of tumor prominence using CDFI in the patient collective of the control group, Figure S2b: Course of tumor prominence using bulbar sonography in the patient collective of the control group.
