# Supplementary material for: Evaluation of Choroidal Melanoma Vascularization by Color Doppler Flow Imaging: An Option for Follow-Up Tumor Control Assessment after CyberKnife®?
Source: Medicina (Kaunas). 2021 May 31;57(6):553. doi: 10.3390/medicina57060553 (PMC8227747; doi:10.3390/medicina57060553)
Supplement: Supplementary file 1 [file medicina-57-00553-s001.zip › medicina-1198594-supplementary.pdf]

Supplemental Figure S1: Course of tumor vascularization during follow-up.

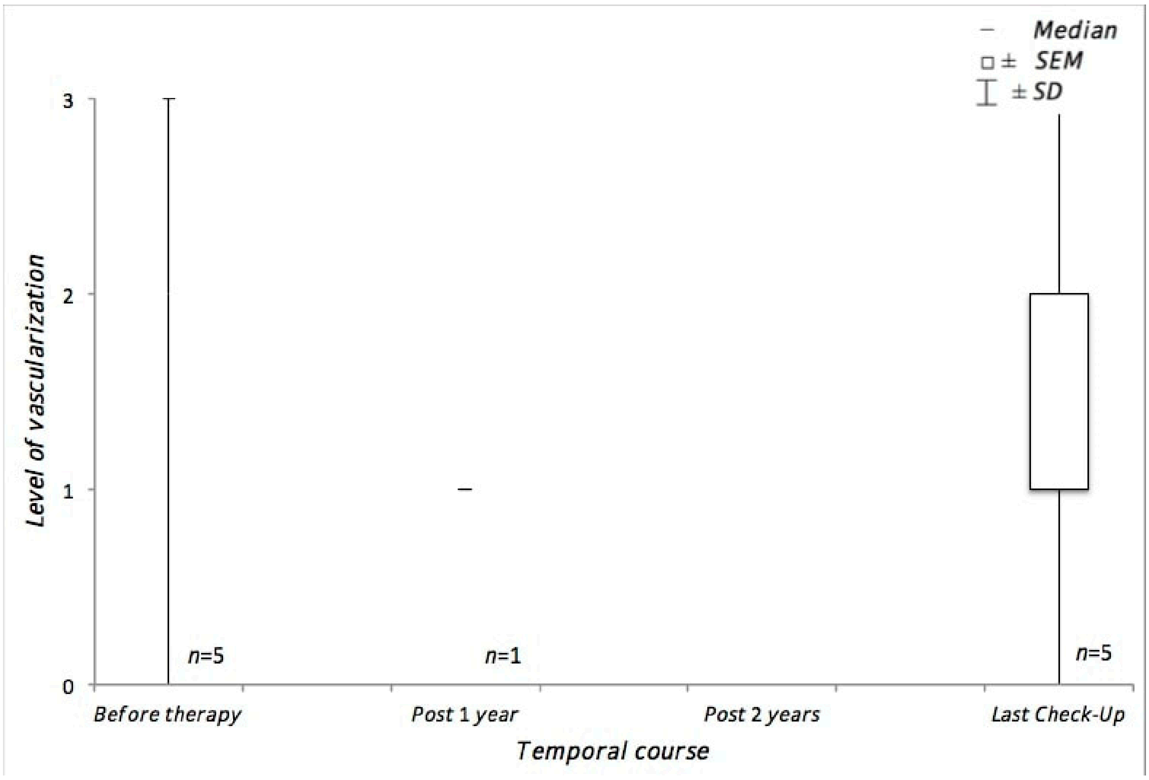

Figure S1. Figure shows the course of tumor vascularization during follow-up in the patient collective of the control group. In this patient population, no significant change in vascularization could be detected. Pre-therapeutically, the mean level of vascularization was 1.8 ± 1.1. Post-therapeutically, the mean level of vascularization could be fixed at 1.6 ± 1.14.

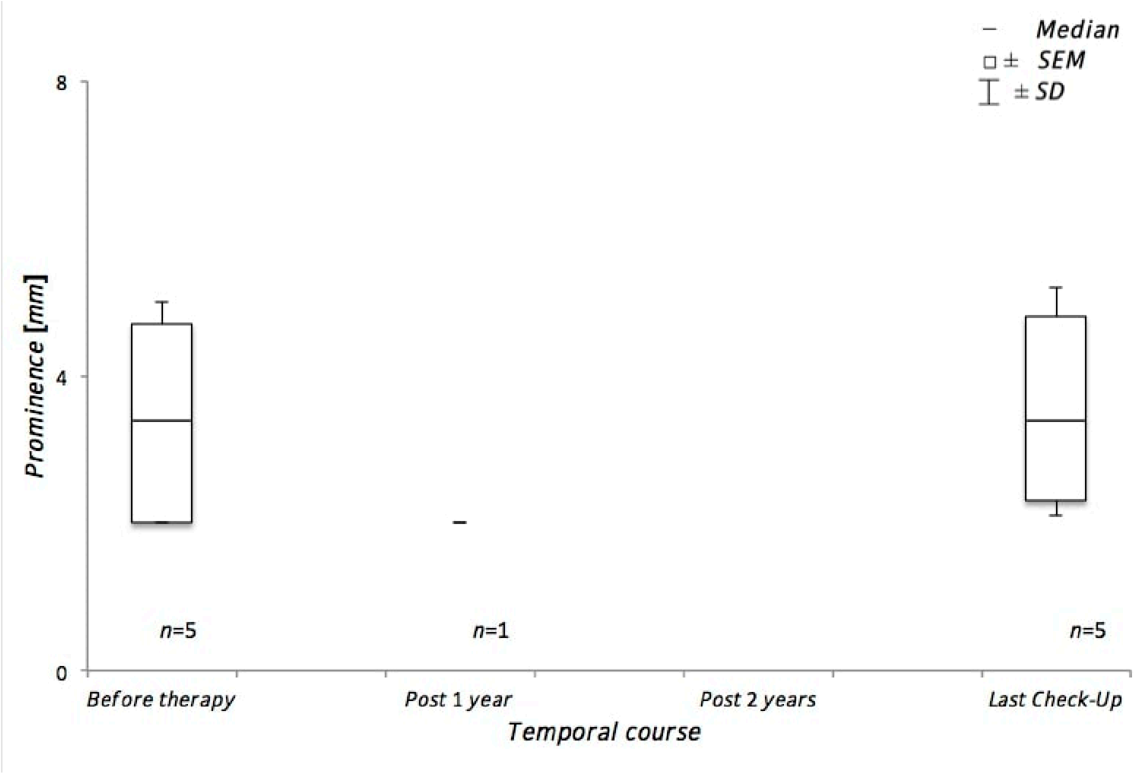

(**a**)

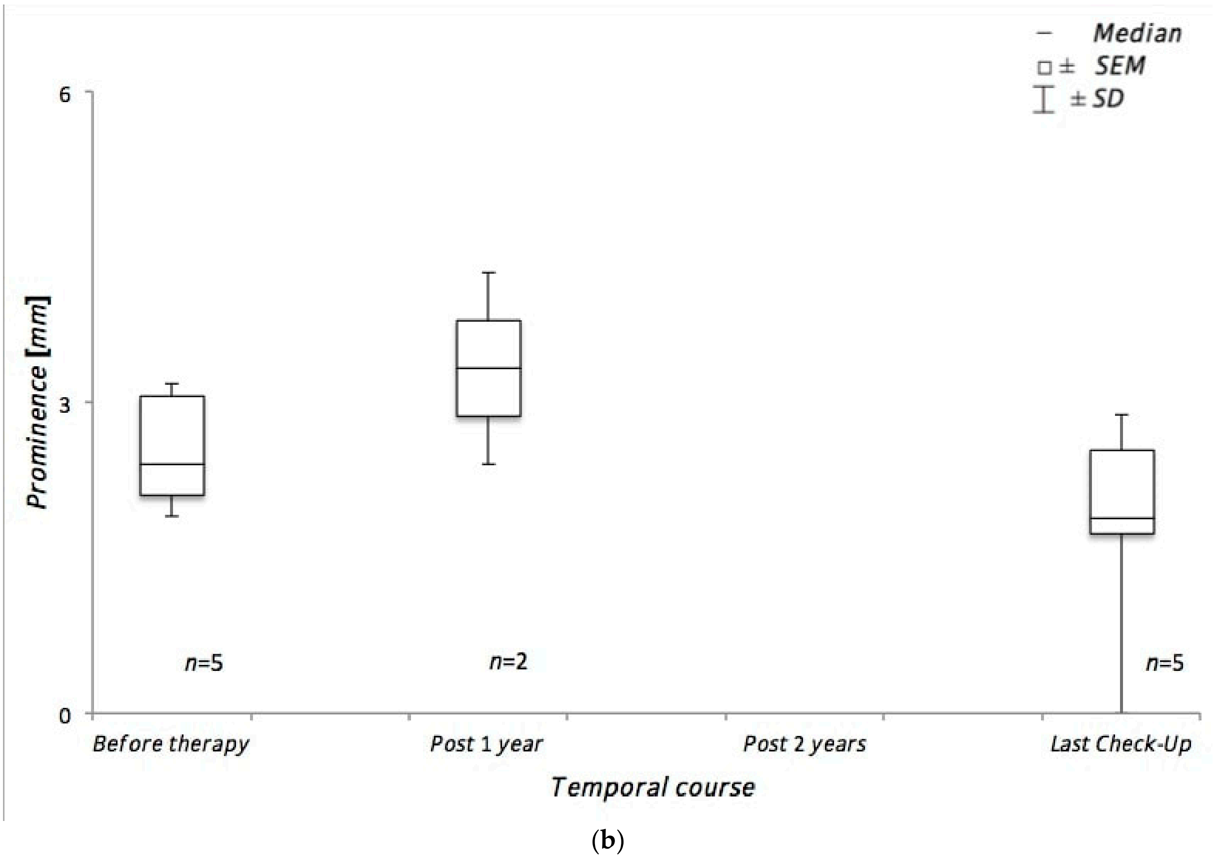

(**b**)

Figure S2. Course of tumor prominence during follow-up. (**a**) shows the course of tumor prominence during follow-up using color Doppler flow imaging in the patient collective of the control group. Before therapy, the tumor prominence had a mean value of 3.42 ±1.43 mm. Post-therapeutically, the tumor prominence showed a mean of 3.56 ± 1.41. (**b**) shows the course of tumor prominence during follow-up using bulbar sonography in the

patient collective of the control group. Pre-therapeutically, the mean tumor prominence was 2.53 ± 0.47 mm. Post-therapeutically, it showed a mean value of 1.81 ±1.11 mm.
